# Structure of serotonin receptors: molecular underpinning of receptor activation and modulation

**DOI:** 10.1038/s41392-021-00668-3

**Published:** 2021-06-18

**Authors:** Markus Zweckstetter, Alexander Dityatev, Evgeni Ponimaskin

**Affiliations:** 1 Translational Structural Biology Group, German Center for Neurodegenerative Diseases (DZNE), Göttingen, Germany; 2 Department for NMR-based Structural Biology, Max Planck Institute for Biophysical Chemistry, Göttingen, Germany; 3 Molecular Neuroplasticity Group, German Center for Neurodegenerative Diseases (DZNE), Magdeburg, Germany; 4 Medical Faculty, Otto-von-Guericke University, Magdeburg, Germany; 5 Center for Behavioral Brain Sciences (CBBS), Magdeburg, Germany; 6 Department of Cellular Neurophysiology, Hannover Medical School, Hannover, Germany

**Keywords:** Structural biology, Molecular neuroscience

**Fig. 1:**
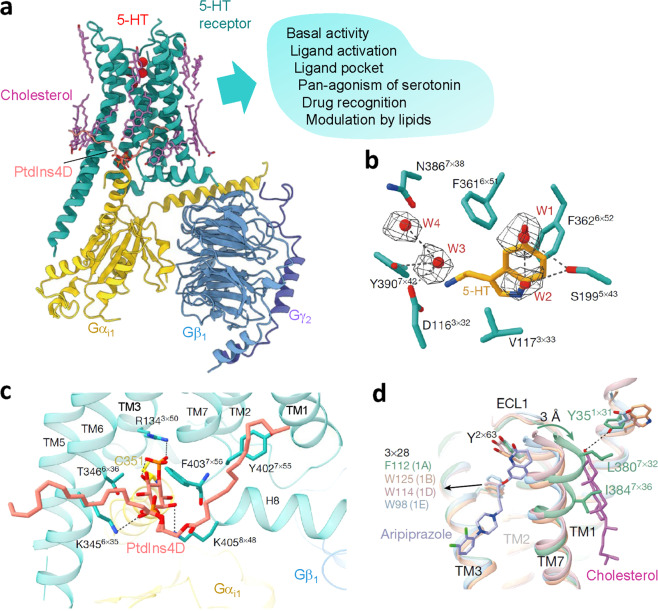
Structural analysis of 5-HT1 receptor activation and modulation by lipids. **a** Structural model of 5-HT1A in complex with Gi and 5-HT in the ligand pocket, and with associated lipids (left). The structure–function relationships discovered on the basis of this and four other resolved structures (right). **b** Water molecules (W1–W4 shown in mesh) in the apo-5-HT1A ligand-binding pocket. Hydrogen bonds are shown with dashed lines. **c** Interactions of PtdIns4P at the 5-HT1A–Gi interface, with interacting residues shown in sticks. Hydrogen bonds are shown with dashed lines. **d** Side view of different TM7 conformations and TM7 interactions with aripiprazole and cholesterol between 5-HT1A, 5-HT1B, 5-HT1D, and 5-HT1E. ECL1, extracellular loop 1. Reprinted by permission from Springer Nature: Nature, Xu et al. © (2021),^1^under exclusive licence to Springer Nature Limited^.^

The structural basis of the regulation of serotonin (5-hydroxytryptamine, 5-HT) receptors by ligands and lipids is only emerging. A recent study by Xu and colleagues^1^ published in *Nature* addresses this issue by resolving five structures of 5-HT_1_ receptor–G protein complexes. These include 5-HT_1A_ in the apo-state, i.e. not bound to a ligand, 5-HT_1A_ and 5-HT_1D_ bound to serotonin or the atypical antipsychotic aripiprazole (for 5-HT_1A_), and 5-HT_1E_ bound to its selective agonist BRL-54443. These high-resolution structures and their comparison enable critical insights into the conformational basis of basal and ligand activation of 5-HT_1_ receptors, the pan-agonism of serotonin, and the mechanisms for modulation of 5-HT_1_ receptors by lipids. Besides, the structural analysis reveals important determinants for ligand selectivity and drug recognition by 5-HT_1_ receptors (Fig. 1a).

The five 5-HT_1_ receptor structures determined by Xu and colleagues^1^ display the canonical seven-TM fold of G-protein-coupled receptors (GPCRs) and are similar to a previously described 5-HT_1B_–G-protein complex.^2^ For the structures in the active state, the cytoplasmic pocket of the 5-HT receptors is open for G-protein binding. The resolved structures provide new insight into the mechanism underlying the high constitutive activity of 5-HT_1A_ receptors: the ligand-binding site in the structure of the 5-HT_1A_ receptor in the apo-state is similar to the structure of the ligand-binding pocket in complex with serotonin. Critical for the structural similarity are water molecules that form hydrogen bonds with residues building the ligand-binding pocket, thus counterfeited polar functionalities of serotonin (Fig. 1b).

Serotonin is an important neurotransmitter of the nervous system. It regulates a broad range of physiological functions including the control of body temperature, appetite, sleep, mood, and pain. Serotonin acts by activating a family of heterogeneously expressed 5-HT receptors, including both GPCRs and ion channels. The 5-HT receptors comprise seven distinct classes based on their structural and functional characteristics. Among them, the inhibitory G-protein-coupled serotonin receptors belonging to the 5-HT_1_ subgroup are key players in the pathophysiology of major depressive disorder, bipolar disorder, schizophrenia, and anxiety disorders^,^^3^^refs herein^. 5-HT_1_ receptors thus have emerged as important therapeutic targets. However, the development of highly selective drug candidates requires a detailed understanding of the molecular underpinnings that determine receptor activation and signaling. Xu and colleagues^1^ provide key insights into these molecular determinants. Across twelve 5-HT GPCRs, which all bind serotonin, only eight out of 22 amino acids are identical in the ligand-binding pocket. Because of this variability in the composition of the ligand-binding pocket, different residues in different 5-HT GPCR subtypes bind serotonin with similar affinities and functional responses, explaining the pan-agonism of serotonin. The cryo-electron microscopy structures further reveal how the 5-HT receptor modulators BRL-54443, aripiprazole, and 5-carboxamidotryptamine (5-CT) act as agonists for different subsets of serotonin receptors. These and other data show that the resolved structures allow to explain the pharmacological properties of available drugs and eventually be used for the design of new potent and specific 5-HT receptor modulators for the treatment of psychiatric diseases.

Structures of GPCRs often display bound phospholipids and cholesterol molecules, and multiple studies demonstrated the functional importance of specific interactions between lipids and GPCRs.^3^ For example, studies of rhodopsin have shown that both polyunsaturated fatty acids and phosphatidylethanolamines can act as ligands interacting with specific sites on the receptor.^4^ Moreover, the discovery of allosteric sites within GPCRs suggests that membrane lipids can bind to allosteric sites acting as ligands further underline the role of membrane–lipid interactions in regulating GPCR dynamics and functions.^4^ Xu et al. identified a phospholipid molecule at the interface between the 5-HT_1A_ receptor and a G-protein (Fig. 1c). The reported electron density is best in agreement with the structure of the phospholipid phosphatidylinositol 4-phosphate (PtdIns4P). The binding of PtdIns4P to the interface might stabilize complex formation and thus contribute to the enhanced activity of the receptor–G-protein complex in the presence of PtdIns4P. Indeed, this was confirmed by mutagenesis and functional studies, which demonstrated that PtdIns4P significantly improves G-protein coupling and GTPase activity, thus boosting the receptor-mediated signaling. Noteworthy, other membrane lipids including phosphatidylethanolamine, phosphatidylcholine, phosphatidylglycerol, and phosphatidylserine also enhanced the 5-HT_1A_ receptor-mediated activation of Gi protein, but to a lower extent, suggesting a specific role of PtdIns4P. Moreover, the interaction of the 5-HT1A receptor with PtdIns4P increased constitutive receptor activity, thus, functioning as a positive allosteric modulator. Two closely related phospholipids, phosphatidylinositol, and phosphatidylinositol 4,5-bisphosphate, could potentially bind the same cavity as PtdIns4P, in agreement with the observation that these two phospholipids also enhance GTP hydrolysis of the receptor–G-protein complex, but these effects were smaller than those obtained for PtdIns4P.

This study also sheds light on the possible role of another major lipid component of the plasma membrane, cholesterol. Although multiple studies demonstrated a vital role of cholesterol in the function and organization of GPCRs, the underlying molecular mechanisms remained largely enigmatic. The structure of 5-HT_1A_ determined by Xu et al.^1^ explains the importance of cholesterol for GPCR activity. Two cholesterol molecules make direct contact with the PtdIns4P molecule at the receptor–G-protein interface. Moreover, one additional cholesterol molecule was found to be inserted into a cleft between the transmembrane (TM) helices TM1 and TM7, potentially contributing to the sculpting of the ligand-binding pocket (Fig. 1d). Receptor-bound cholesterol thus might directly contribute to the high affinity of aripiprazole for 5-HT_1A_ receptors. However, additional experiments, for instance, using cholesterol depletion/substitution in combination with cryo-electron microscopic analysis, are required to dissect the contribution of bound cholesterol to the structure of the ligand-binding pocket of 5-HT receptors.

Previous studies revealed a crucial role of 5-HT_1A_ receptor palmitoylation at its C-terminal cysteine residues Cys417 and Cys420 for 5-HT_1A_/G_i_-protein coupling and effector-mediated signaling.^3^ An important 5-HT_1A_ receptor palmitoyl acyltransferase is the zinc finger DHHC-type palmitoyltransferase 21 (ZDHHC21) with epigenetic downregulation of ZDHHC21 linked to major depression and suicide.^3^ Receptor palmitoylation targets it to cholesterol-rich membrane microdomains. It would therefore be highly interesting to further study the synergistic effects of cholesterol and palmitoylation on the structure and function of known palmitoylated serotonin receptors, including 5-HT_1A_, 5-HT_4,_ and 5-HT_7_.

In conclusion, the study by Xu and colleagues^1^ represents an important step toward deciphering mechanisms of 5-HT receptor-mediated signal transduction at the molecular level. However, the reported cryo-electron microscopy structures only represent a small fraction of the ensemble of conformations present in the analyzed samples. Indeed, a key property of GPCRs is their pliability.^5^ A full understanding of the molecular basis of 5-HT-mediated signaling is therefore likely to require elucidation of the dynamics of 5-HT receptors and their complexes.
